# A Novel Approach to Boosting Glutathione via Iontophoresis

**DOI:** 10.7759/cureus.17803

**Published:** 2021-09-07

**Authors:** Sajad Zalzala

**Affiliations:** 1 Integrative/Complementary Medicine, AgelessRx, Ann Arbor, USA

**Keywords:** glutathione, aging, longevity, supplementation, iontophoresis

## Abstract

Two cases (a 73 and a 67-year-old) with low serum reduced glutathione (GSH) were supplemented with GSH using IontoPatch™ (IontoPatch, St. Paul, USA) to determine whether GSH serum levels could be restored to within the reference range using this technology. A 1 mL dose of a 200 mg/mL saline solution of GSH was added to the patch’s negative electrode for each treatment. The patch was applied on the upper arm’s skin and was worn for six consecutive days for at least four hours each day. Serum levels of GSH were assessed at baseline and days 7 and 23 after treatment was initiated. In both cases, serum GSH levels increased after seven days of treatment (64.4 and 21.8%). Serum GSH levels then decreased between days 7 and 23 to 44.5 and 17.2% above baseline. There were no adverse events reported in either case. More extensive studies should be conducted to determine the pharmacokinetics, safety of long-term supplementation, and supplementation health benefits.

## Introduction

Glutathione (GSH) is the most abundant exogenous antioxidant in human cells and is found in both the reduced (GSH) and oxidized (glutathione disulfide (GSSG)) forms. GSH is a tripeptide comprised of cysteine, glutamic acid, and glycine. Depletion of GSH may occur when the cellular levels of these components are limited or when demand is high. GSH depletion increases the cell’s susceptibility to damage to DNA, RNA, lipids, and proteins caused by oxidants and reactive metabolites.

Reactive oxygen species (ROS) produced in the body can promote oxidative damage to cells and may cause genomic instability and mitochondrial dysfunction, two hallmarks of aging [1]. The concentration of GSH has been shown to decrease with aging [2,3], resulting in reduced antioxidant activity in cells. Consequently, lower GSH levels have been associated with an increased risk of aging-associated diseases, such as cardiovascular diseases, arthritis, macular degeneration, Alzheimer’s disease, Parkinson’s disease, and diabetes [3-7]. Relatively higher blood levels of GSH, on the other hand, are associated with improved physical and mental health in older individuals [5,8] and with a reduced risk of developing Alzheimer’s disease [9]. Supplementation of GSH may, therefore, protect against age-related morbidity and mortality.

Supplementation of GSH has its challenges as it is a water-soluble peptide that has to cross the hydrophobic plasma membrane and the half-life in plasma is short at 1.6 minutes [10]. Oral supplementation additionally requires a formulation that prevents acidic breakdown in the stomach environment and can be achieved through liposomal formulations [11]. However, indirect oral supplementation is possible by intake of whey protein. In recent years, intravenous (IV) supplementation has become a popular method to restore GSH levels. It is an effective method [12] but has its limitations as it is only accessible in a specialty clinic setting and is expensive and inconvenient for patients.

Two aging patients with low serum GSH levels were supplemented with GSH in our clinic using a non-invasive drug delivery device, the IontoPatch™ (IontoPatch, St. Paul, USA), to deliver GSH through the skin. The IontoPatch™ technology uses bipolar electric fields, iontophoresis, to deliver molecules across the skin into the underlying tissue. Iontophoresis is widely used in physical therapy for localized treatment of pain and inflammation [13].

## Case presentation

Two healthy older participants were found to have abnormally low serum GSH levels measured. The first case was a 73-year-old Caucasian non-smoker male with a history of coronary artery disease, hypothyroidism, hypertension, and hyperlipidemia. The case was on the following medication: levothyroxine 88 mcg, Plavix 75 mg, allopurinol 300 mg, and atorvastatin 80 mg. Additionally, the patient uses several supplements, including vitamin C, D, arginine, magnesium, berberine, B-complex, and zinc. During blood tests and GSH treatment, the patient was in a normal state of health. At initial blood draw, total GSH was below the reference range (544-1228 μM) at 393 μM, interleukin 6 (IL-6) was within the normal range (<5.00 pg/mL) at 1.68 pg/mL, and tumor necrosis factor-alpha (TNF-α) was also within the normal range (0.56-1.40 pg/mL) at 0.71 pg/mL.

The second case was a 67-year-old Caucasian non-smoker female with a history significant for ductal carcinoma in situ (DCIS) breast cancer and pre-diabetes. The patient was on the following medication: Arimidex 1 mg, metformin 500 mg. Additionally, she takes a vitamin D supplement. The initial serum level of GSH was 482 μM (below the reference range: 544-1228 μM), and IL-6 and TNF-α levels were within the normal range at 4.95 and 0.63 pg/mL, respectively. 

Both patients were placed on GSH supplementation using the IontoPatch™ to increase serum GSH levels. The IontoPatch™ was obtained from IontoPatch (St. Paul, MN, USA), and a 200 mg/mL buffered GSH solution was obtained from Archway Apothecary (Covington, LA, USA). Before each use, 1 mL (20 drops) of GSH solution was applied to the negative electrode of the patch, 1 mL (20 drops) of saline solution was applied to the positive electrode. The IontoPatch™ was worn for six subsequent days for at least four hours each day and contained 200 mg GSH placed on the patch’s negative electrode. The IontoPatch was worn on the upper arm using the left and right sides alternately every day. On day 6 of treatment, the patch was removed in the evening.

Blood was drawn on day 7, approximately 16 hours after removing the last patch, and 16 days after removing the patch. Blood was drawn at the same time of day (noon) at each time point (including the baseline draws), with the patient in a non-fasting state. Serum was assessed for total glutathione at both after treatment timepoints, and serum IL-6 and TNF-α were assessed on the day after removal of the IontoPatch. Quest Diagnostics performed all blood tests; GSH: test code 90379, IL-6: test code 34473, TNF-α: test code 34485. 

**Figure 1:**
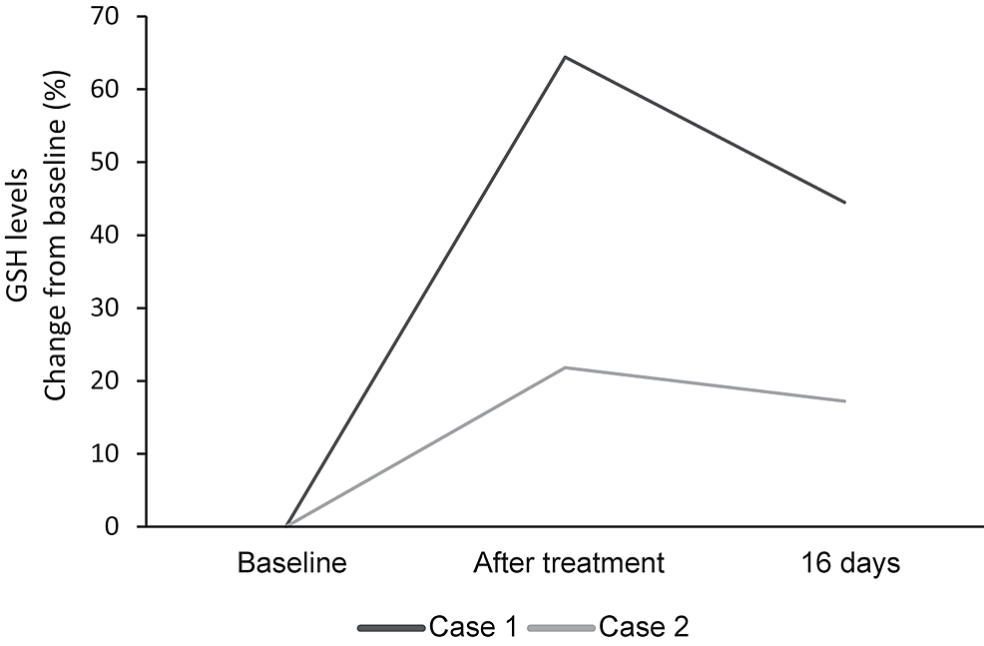
Changes in GSH serum levels in percentage from baseline. GSH levels of both cases increased from baseline after treatment and this increase was still present 16 days after treatment. GSH: glutathione.

Following treatment, the first patient's GSH level increased into the reference range at 646 μM, an increase from baseline of 64.4% (Figure 1). IL-6 (4.18 pg/mL) and TNF-α (0.76 pg/mL) remained within the normal range. Sixteen days following treatment, total GSH levels declined by 12.1% to 568 μM, which remained within the reference range and was 44.5% higher than the baseline level. The patient did not experience any adverse events from the patch or the treatment but reported increased energy levels.

Following treatment, the second patient's GSH level increased by 21.8% to 587 μM following the treatment interval. Sixteen days following the treatment interval the second patient's GSH level remained 17.2% higher than baseline at 565 μM (Figure 1). IL-6 and TNF-α levels remained within normal ranges after treatment (IL-6: 4.18 pg/mL; TNF-α: 0.76 pg/mL). The patient did not report any adverse effects from treatment but did report a subjective increase in energy.

## Discussion

The two elderly cases investigated in this report had GSH serum levels below the reference range on initial blood testing, consistent with the literature suggesting that GSH levels decline with age [2]. We were able to increase their total GSH serum to values within the reference range for healthy adults using the IontoPatch™ for GSH supplementation. Blood levels remained within the reference range up to 16 days after treatment, suggesting treatment can be performed intermittently. Further analysis of dose-response in a larger patient population would help define the best treatment schedule for aging patients.

Reductions in GSH levels have been detected in aging populations [2] and patients with cystic fibrosis [14], Parkinson’s disease [15], and those infected with HIV [16]. In the aging population, GSH levels are lower due to a reduction in synthesis and can be restored with supplementation, resulting in a reduction in plasma markers of oxidative stress [2]. Due to its various roles, GSH is essential for cellular and tissue homeostasis, cell proliferation, and immune function [4,17]. Therefore, supplementation in individuals with low GSH levels could potentially have various health benefits. In this case report, we present two cases in which we supplemented GSH with the IontoPatch to increase serum levels, and therefore, we did not assess health benefits. However, the two cases both reported increased energy levels. In more extensive future studies, the health benefits for aging individuals should be assessed.

Since GSH levels are essential for immune function and high levels have been shown to protect against influenza infection [4,18], we assessed the cytokines IL-6 and TNF-α in serum after treatment. We did not detect any changes in the serum levels of these cytokines in the two patients. A GSH supplementation study in patients with liver disease suggested that monocyte cytokine production, including IL-6 and TNF-α, decreased [19]. Furthermore, GSH levels may affect nuclear factor kappa-light-chain-enhancer of activated B cells (NF-κB) activation and change the immune response from Th1-driven to Th2-driven, which is seen in aging populations. The lack of changes in cytokine levels detected here may be due to the serum assessment, and we only measured it at the time point directly after treatment. A more comprehensive cytokine and immune profiling would be of interest to investigate the effects of GSH supplementation on the immune response in aging individuals.

The supplementation via the iontophoresis method would allow for simultaneous supplementation of two molecules, one on the positive and one on the negative electrode. A potential addition to the GSH patch would be the supplementation of nicotinamide adenine dinucleotide (NAD^+^) on the positive electrode. NAD is a coenzyme in redox reactions and a signaling molecule found in human cells that has, like GSH, been shown to decrease with age and be associated with age-associated diseases [20]. Supplementing both simultaneously, using the same patch for iontophoresis would make these therapies more accessible and potentially synergistic in functionality.

Limitations of this study include the small sample size (N = 2), the lack of a control arm, that we did not assess clinical outcome measures other than blood values, and we did not determine the long-term status of GSH after a single treatment. The results presented here provide a rationale for conducting a larger study to assess the long-term effects and intermittent dosing schedule.

## Conclusions

We show here that IontoPatch™ technology can be used to supplement GSH in aging individuals with low serum GSH levels. Supplementation using iontophoresis did not cause any apparent side effects. Serum GSH could be supplemented to levels within the reference range, providing a healthy dosage for those with deficiencies. Larger studies should be conducted to determine the pharmacokinetics, safety of long-term supplementation, and health benefits of supplementation, as well as an assessment on how this method of supplementation compares to other methods of supplementation, such as indirect oral and IV supplementation.
